# Mitraclip Versus Medical Therapy or Surgery in Patients With Mitral Regurgitation: Long-Term Outcomes Determined by the Reconstruction of Individual Patient Data

**DOI:** 10.7759/cureus.60204

**Published:** 2024-05-13

**Authors:** Andrea Messori, Valeria Fadda, Melania Rivano, Sabrina Trippoli

**Affiliations:** 1 Health Technology Assessment (HTA) Unit, Regione Toscana, Florence, ITA; 2 Pharmacology and Therapeutics, Ente di Supporto Tecnico Amministrativo Regionale (ESTAR), Florence, ITA; 3 Clinical Oncology Pharmacy Department, Binaghi Hospital, Cagliari, ITA

**Keywords:** mitral regurgitation, mitraclip, ipdfromkm method, individual patient data reconstruction, survival gain

## Abstract

Although MitraClip has been studied in numerous trials, its evidence in the long term is based on a few original studies. We used an original technique of evidence synthesis to review long-term comparative trials evaluating MitraClip. We searched the PubMed database to select long-term comparative trials of MitraClip. The endpoint was all-cause mortality (minimum follow-up, one year). Included trials were analyzed using the IPDfromKM (reconstruct Individual Patient Data from published Kaplan-Meier survival curves) method to reconstruct individual patient data from Kaplan-Meier curves. Standard survival statistics were used to interpret these long-term efficacy data. The survival benefit per patient was estimated from the restricted mean survival time (RMST). Six comparative studies of MitraClip were included; 973 patients were treated with MitraClip (six arms), 717 with medical therapy (five arms), and 80 with surgical repair or replacement (one arm). In our main analysis, the outcomes observed in patients treated with MitraClip were significantly better than those of medical therapy (hazard ratio for all-cause mortality, 0.5276; 95% confidence interval, 0.4412 to 0.6309; p < 0.001); the number of patients treated with surgery was too small to make reliable comparisons. Median survival was 30.4 months for medical therapy versus not reached for the other two groups. RMST was 43.931 and 33.756 months for MitraClip and controls, respectively, yielding a gain per patient of 10.17 months (95% confidence interval, 7.47 to 12.88). In our simplified cost-effectiveness evaluation, a gain of approximately 10 months per patient compared favorably with the device cost. Our analysis provided an original interpretation of the long-term evidence available on MitraClip.

## Introduction and background

MitraClip is a well-known therapeutic option for transcatheter valve repair in patients with mitral regurgitation. The advantages of MitraClip over medical therapy have been demonstrated in some important pivotal studies, but conflicting results have also been reported. 

The aim of this study is twofold: 1) to compare the efficacy of MitraClip with medical therapy or surgery based on all published literature to date and 2) to perform this comparison using an innovative method of evidence-based analysis (the so-called IPDfromKM (reconstruct Individual Patient Data from published Kaplan-Meier survival curves) method), which is increasingly being used to study cardiological issues.

The evidence for the long-term efficacy of MitraClip in patients with mitral regurgitation [1] comes almost entirely from three well-known pivotal randomized trials (Endovascular Valve Edge-to-Edge Repair Study II (EVEREST-II) [2], Cardiovascular Outcomes Assessment of the Mitra Clip Percutaneous Therapy for Heart Failure Patients with Functional Mitral Regurgitation (COAPT) [3], and Multicentre Study of Percutaneous Mitral Valve Repair MitraClip Device in Patients With Severe Secondary Mitral Regurgitation (MITRA-FR) [4]) and a small number of non-randomized controlled studies. Irrespective of the presence of randomization, all control groups in these trials were treated with standard medical therapy, with the sole exception of the EVEREST-II trial, in which controls received surgical valve repair. On the other hand, the IPDfromKM method represents an interesting new technique for evidence-based analysis due to its ability to reconstruct individual patient data from Kaplan-Meier plots [5-11], especially when the focus is on the long term.

This review has been published in part as a reprint [12].

## Review

Methods

Study Design

Our review identified pertinent studies through a standard PubMed search. These studies were then subjected to a reconstruction of individual patient data, which was performed using the IPDfromKM method. Reconstructed patients were then included in a comparative survival analysis to evaluate MitraClip versus medical therapy or surgery in terms of long-term effectiveness. The heterogeneity of this clinical material was assessed through methods accounting for the length of follow-up. Finally, the survival gain determined by MitraClip was quantified to generate a preliminary estimate of its cost-effectiveness.

Literature Search

We identified eligible articles using a standard PubMed search (search term: "Mitraclip" combined with setting the filter to "clinical trial"). Among these articles, pertinent trials were then selected based on the criteria of comparative design, use of MitraClip in at least one arm, all-cause mortality as the endpoint, and availability of a Kaplan-Meier survival graph. The flow of article selection was managed through the Preferred Reporting Items for Systematic Reviews and Meta-Analyses (PRISMA) algorithm [13]. Our analysis of included trials was aimed at combining the survival information reported in the original reports into a pooled survival assessment evaluating long-term outcomes, statistical significance, and heterogeneity between trials.

Reconstruction of Individual Patient Data

The IPDfromKM method [5,6] (or Shiny method), implemented with the use of artificial intelligence, is a relatively new evidence-based method, which has been used mainly to study anticancer agents [7]. More recently, however, the application of the IPDfromKM method has been extended to the field of cardiology [8-11]. For these reasons, we chose the IPDfromKM method to conduct an updated analysis of the evidence on MitraClip versus medical therapy, with a particular focus on clinical trials based on long-term follow-up. While some standard meta-analyses have been performed on this topic [1], long-term survival outcomes can be examined more accurately when results are presented using a Kaplan-Meier curve rather than a hazard ratio. Importantly, reconstructed patient-level data are known to perform well in these circumstances [7-11]. According to recommendations on using the IPDfromKM method, Webplotdigitizer was used to digitalize Kaplan-Meier graphs; then, the IPDfromKM online software was run to reconstruct individual patient data from patient arms reported in included studies.

Statistical Analysis

Each arm of the included trials was subjected to a standard IPDfromKM analysis [5,6]. Then, in our analysis of pooled trials, the three treatments (MitraClip vs. medical treatment or surgical intervention) were compared based on the endpoint of all-cause mortality. All analyses were performed on reconstructed individual patient data. The statistical comparisons were performed according to the hazard ratio (HR); 95% confidence intervals (CI) were determined where appropriate.

Regarding the design of our statistical analysis, in running the IPDfromKM method, all patient arms treated with MitraClip were pooled together to generate a single survival curve. Likewise, all patient arms treated with medical therapy were pooled into a single arm. Only a single patient arm was available for the surgical option. Finally, the three curves of reconstructed patients for MitraClip, medical therapy, or surgery were compared by standard survival statistics (i.e., Cox model with HR estimation). For this purpose, four packages ("survival," "survminer," "survRM2," and "readxl") of the R-platform [14] were used. Censored patients were managed through standard methods of time-to-event statistics. The survival gain per patient was estimated as the difference in the restricted mean survival time (RMST) [15]. Regarding heterogeneity, this parameter was assessed in two separate analyses according to the typical approach required by the IPDfromKM method [6-11], which determines the likelihood ratio test and Wald’s test.

Articles selected through our literature search

**Figure 1:**
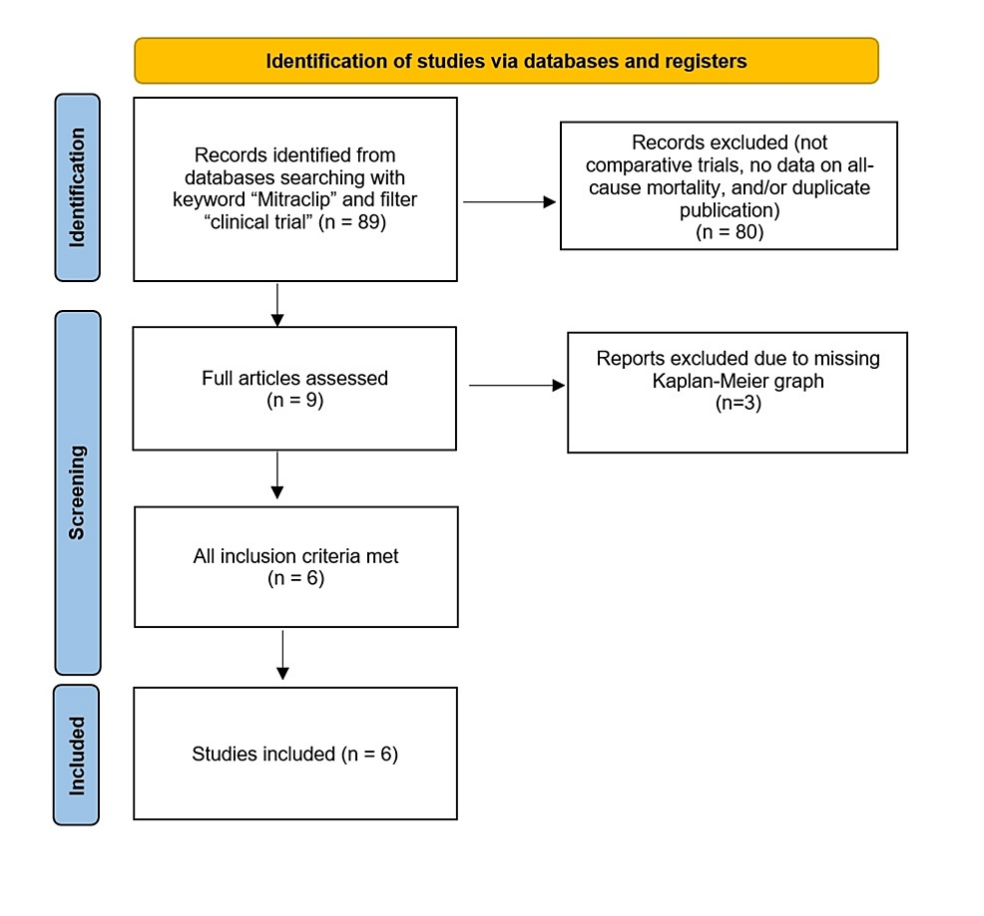
Literature search according to the PRISMA flowchart. PRISMA: Preferred Reporting Items for Systematic Reviews and Meta-Analyses

Figure 1 shows the selection process based on the PRISMA algorithm, which identified a total of six comparative studies.

Three randomized studies (EVEREST-II [2], COAPT [3], and MITRA-FR [4]) and three non-randomized studies [16-18] were included in our analysis. Both COAPT and MITRA-FR compared MitraClip with medical therapy, while EVEREST-II compared MitraClip with surgical intervention. The results of COAPT and MITRA-FR were conflicting because the former found the superiority of MitraClip over medical therapy, but the latter did not find any advantage. One limitation of the EVEREST-II trial is that it adopted a 2:1 randomization that led to enrol relatively few patients in the surgical arm. All three non-randomized trials (published by Armeni et al. [16], Asgar et al. [17], and Giannini et al. [17]) compared MitraClip with medical therapy; their results were in favor of the MitraClip option.

By contrast, the following three non-randomized studies [19-21] were excluded because a Kaplan-Meier graph was not reported: i) Hubert at al. [19] (n = 37 for MitraClip vs. n = 19 for medical therapy) in which the follow-up was six months; ii) Papadopoulos et al. [20] (n = 86 for Mitraclip vs. 28 for medical therapy; follow-up of one year); iii) Krawczyk-Ożóg A et al. [21] (n = 10 for MitraClip vs. 23 for medical therapy; follow-up of eight months). It should be noted that these three trials [19-21] either included small populations of patients or had a short follow-up; furthermore, their outcomes were similar to those of the six included studies, so the impact of their exclusion was likely to be negligible.

The main characteristics of the six included trials are presented in Table 1. In these trials, 973 patients were treated with MitraClip (six arms), 717 with medical therapy (five arms), and 80 with surgical repair or replacement (one arm). 

**Table 1:** Synopsis of the three pivotal trials comparing transcatheter replacement with surgical replacement or medical treatment. The endpoint of these trials is all-cause mortality. Abbreviations: MitraClip, transcatheter aortic valve replacement; EVEREST-II, COAPT, Cardiovascular Outcomes Assessment of the Mitra Clip Percutaneous Therapy for Heart Failure Patients with Functional Mitral Regurgitation, MITRA-FR, Multicentre Study of Percutaneous Mitral Valve Repair MitraClip Device in Patients With Severe Secondary Mitral Regurgitation

Trial (acronym or first author)	Comparison	Follow-up	MitraClip arm		Medical therapy arm		Surgery arm	
			N° of patients	N° of events	N° of patients	N° of events	N° of patients	N° of events
EVEREST-II [2]	Mitraclip vs. surgical valve replacement	5 years	178	32	-	-	80	15
COAPT [3]	Mitraclip vs. medical treatment	5 years	302	162	312	189	-	-
MITRA-FR [4]	Mitraclip vs. medical treatment	2 years	151	53	152	52	-	-
Armeni [16]	Mitraclip vs. medical treatment	1 years	232	20	151	27	-	-
Asgar [17]	Mitraclip vs. medical treatment	1 years	50	11	42	18	-	-
Giannini [18]	Mitraclip vs. medical treatment	3 years	60	23	60	40	-	-

Main analysis with the generation of time-to-event curves based on pooled treatment groups

**Figure 2:**
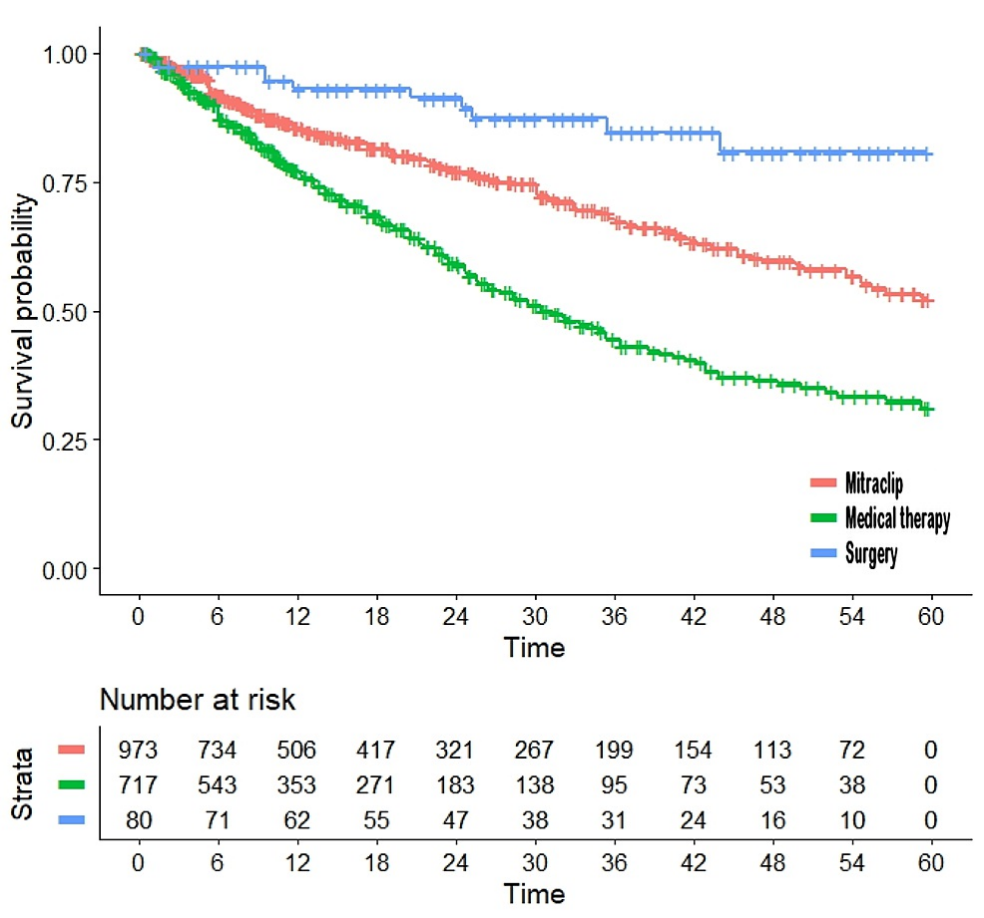
Main analysis: outcomes observed in the patients treated with MitraClip (six arms; n = 973; in red), medical therapy (five arms; n = 717; in green), or surgery (one arm; n = 80; in blue). Pooled data drawn from the six comparative studies reported in Table 1; patients at risk are reported below the Kaplan-Meier graphs. Endpoint, all-cause mortality; time in months.

In our main analysis, three curves were generated for MitraClip, medical therapy, and surgery (Figure 2).

The hazard ratio for the comparison of MitraClip versus medical therapy was 0.5276 (95% CI, 0.4412 to 0.6309; p < 0.001). The number of patients treated with surgery was too small to make reliable comparisons. Median survival was 30.4 months in the medical therapy group and was not reached in the other two groups. 

Heterogeneity analysis

**Figure 3:**
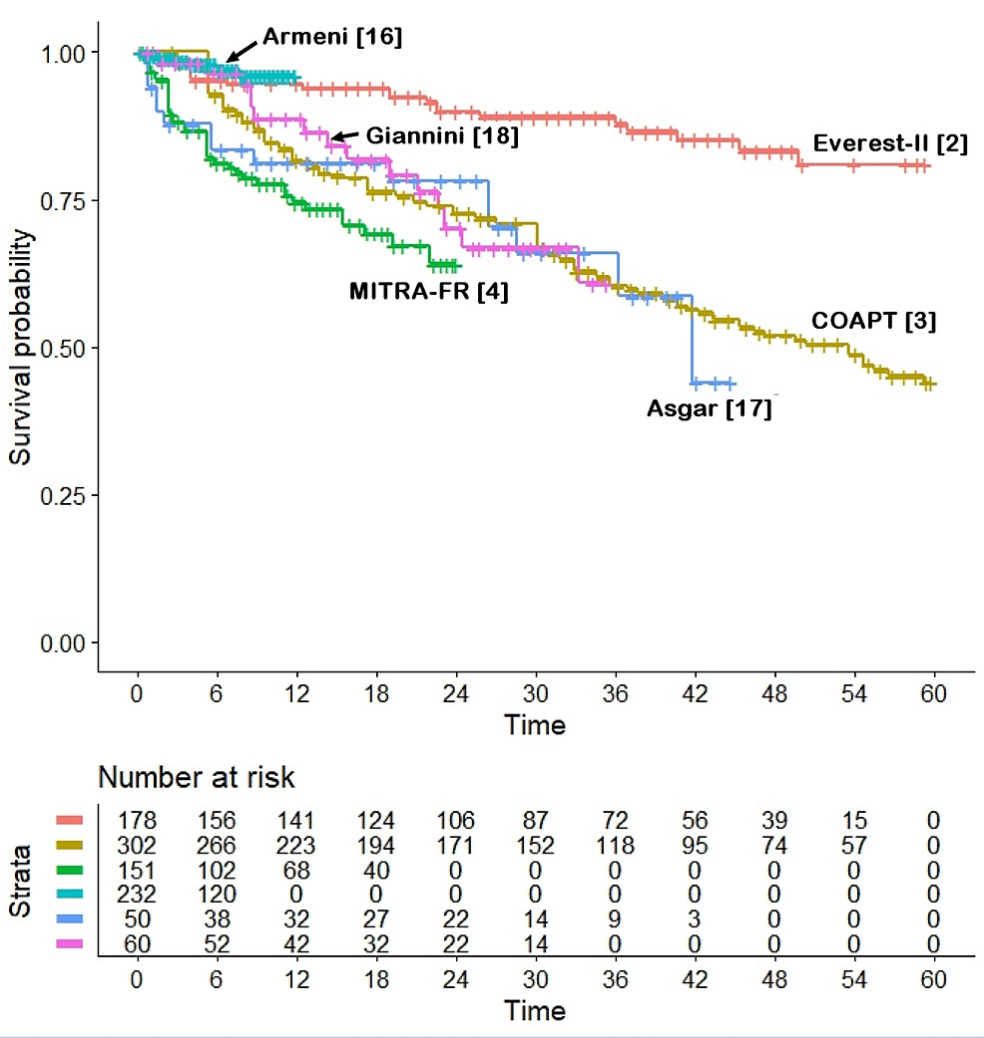
Heterogeneity of outcomes for 973 patients treated with Mitraclip. Endpoint, all-cause mortality; time in months. Trials are identified according to their acronym or first author; patients at risk are reported below the Kaplan-Meier graphs.

**Figure 4:**
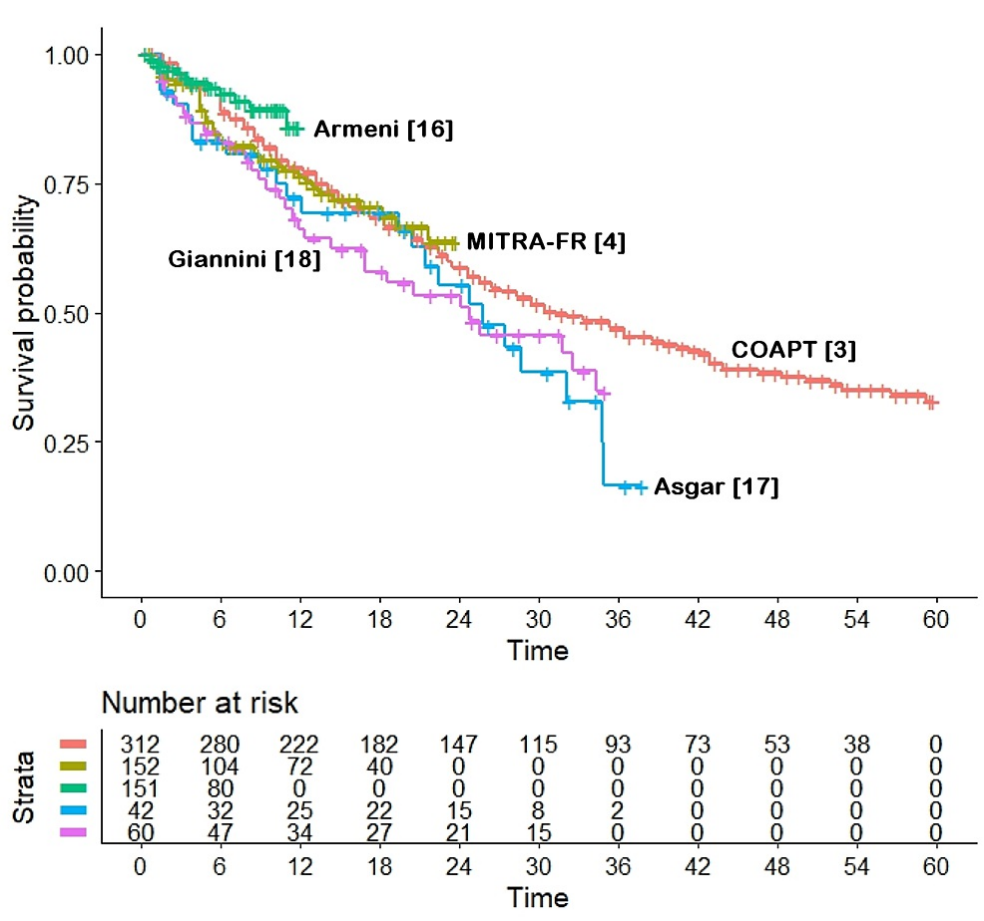
Heterogeneity of outcomes for 717 patients treated with medical therapy. Endpoint, all-cause mortality; time in months. Trials are identified according to their acronym or first author; patients at risk are reported below the Kaplan-Meier graphs.

Our assessment of heterogeneity (Figures 3, 4) provided important findings to improve the interpretation of these results.

In the analysis including the six MitraClip arms (Figure 3), the degree of heterogeneity was extremely high likelihood ratio test, 63.4, df 5, p < 10^-11^; Wald test, 49.37, df 5, p < 10^-8^). A simple visual inspection of the six survival curves (Figure 3) confirms this finding: for example, the HR for the comparison of the MitraClip arm of the MITRA-FR trial versus the MitraClip arm of the COAPT trial showed an astonishing HR of 4.619 (95% CI, 2.514 to 8.489, p < 10^-6^). Interestingly, in a heterogeneity analysis in which the MITRA-FR trial was excluded, the overall level of heterogeneity was borderline, and only the EVEREST-II trial differed significantly from the other trials (data not shown). On the other hand, the degree of between-trial heterogeneity was much lower in the five arms treated with medical therapy (Figure 4), with a borderline statistical significance (likelihood ratio test, 9.26; df 4; p = 0.05; Wald test, 9.17, df 4, p = 0.06).

Estimation of RMST

The RMST values estimated from the pooled curves of MitraClip and medical therapy were 43.93 months (95% CI, 42.22 to 45.65) and 33.76 months (95% CI, 31.66 to 35.85), respectively. This resulted in a survival gain of 10.17 months per patient (95% CI, 7.47 to 12.88, p = 0.01), i.e., 0.8475 years per patient. Assuming a device cost of €23,069, as reported by Armeni et al. [15], this results in a preliminary cost-effectiveness ratio of €27,220 per year of life gained, confirming the favorable profile reported in previous economic literature [16]; this ratio remains virtually unchanged (€25,958 per year of life gained) if one uses the current Italian price of the device (€22,000). It should be emphasized that this economic analysis is highly simplified, as some potential determinants of cost-effectiveness (e.g., implantation cost, human resources to monitor the patient over the years, impact on quality of life) were not taken into account. 

Interpretation of findings

The use of an innovative method of evidence-based analysis gives our research both originality and methodological strength. In fact, this is the first application of the IPDfromKM method among the numerous reports published to date on MitraClip. The overview of the evidence on this complex therapeutic issue (as reported in Figures 2, 3, 4) has the advantage of summarizing the main points of interest on this topic based on detailed follow-up information, including the unexpected difference between the MitraClip arms of the COAPT and MITRA-FR trials. The reasons for the opposite results of these two randomized trials have been discussed in numerous publications [22-25]. The heterogeneity analyses performed in our study provide some insights to better interpret these conflicting results, but the question remains open. In fact, the controls of the MITRA-FR trial show a survival pattern that is not too different from that of the other four trials (which do not include COAPT simply because this trial did not include any medical therapy arm). This can be clearly seen in Figure 4 (representing the heterogeneity across control arms given medical therapy), where the MITRA-FR trial appears to be similar to the others, even though its follow-up is limited to two years. Since these data of the MITRA-FR trial indicate that the baseline prognostic characteristics of its controls were in line with those of the other control groups, there is no simple explanation for the poor survival observed in the MitraClip arm of the MITRA-FR trial.

## Conclusions

The main result of this study is the efficacy comparison generated by our main analysis, in which an interesting feature is the use of the IPDfromKM method. According to our results, the long-term efficacy profile of MitraClip is well established and its cost-effectiveness profile is also acceptable based on current willingness-to-pay benchmarks. The surprisingly unfavorable results reported in the French study were also confirmed, but - even in the light of the overall international evidence on Mitraclip - this finding remains unexplained.
